# 

**Published:** 1898-07

**Authors:** 


### INDEX: VOLUME XIV, JULY, 1898, JUNE, 1899.

ORIGINAL CONTRIBUTIONS—

A Case of Inversion of the Uterus. By F. A. Schmitt, M. D., La Grange, Texas ....................................415
Complete Paralysis—Sensation and Motion—Due to Malarial Toxæmia—Report of a Case—Recovery. By F. R. Collard, M. D., Wheelock, Texas ....................................690
A Confederate Veteran's Thoughts on the Blue and the Grey. By
[illegible]pendicitis—A Possible Cause—The Use of the Ligature—Is it Necessary. By Wm. T. Oppenheimer, M. D .......................... 25
Asthma. By H. B. Hill, M. D., Austin ..........................349
Cæsarian Section—with Recovery of Mother, and Child Saved. By James Byars, M. D., Columbus, Texas ..........................554
Catheters and Cystitis. By R. N. Mayfield, M. D., New York.........417
Chronic Cystitis of the Aged. By I. J. Jones, M. D., Austin..........355
Clinical Report on the Therapeutic Value of Glyco-Thymoline........534
Complete Prolapsus of Uterus—Lacerated Perineum—A Comparatively New Operation Performed, with Report of Two Cases. By J. S. Zvesper, M. D., Ammansville, Texas ..............................413
Continued Malarial Fever. By W. B. Campbell, M. D ............... 27
Dental Notes. By F. S. Caspar, D. D. S., Austin, Texas............625
Diphtheria and Membraneous Croup; Their Differential Diagnosis and Treatment. By Wilson T. Davidson, M. D ....................... 13
Eclampsia. By M. L. Langford, M. D., Baileyville, Texas...........289
Electricity in the Phenomena of Animal Life. By M. Ernest Solvay...477
Etiology and Treatment of Endometritis. By J. S. Watson, M. D., Manor, Texas ....................................608
Internal Hemorrhage. By F. Paschal, M. D., San Antonio, Texas....248
Medical and Surgical Practice During the Santiago Campaign. By William F. James, M. D., San Antonio, Texas....................599
Operation for Ovarian Cyst in an Eleven Year Old Girl. By R. J. Alexander, M. D., Troy, Texas....................................163
Otitis. By J. M. Woodson, M. D., Temple, Texas....................294
Photo-Micrography as Related to Medical and Scientific Research. By Rudolph Menger, M. D., San Antonio, Texas..................671
Pneumonia. By R. W. Wallis, M. D., Rockdale, Texas...............298
Relation of Physicians to the Public—Medical Jurisprudence. By Hon. R. L. Henry, M. C., Waco, Texas..........................682
Report of Laparotomy for Obstruction of Bowels. By F. R. Collard, M. D.................................................... 22
Report of Three Cases of Aoritic Aneurism. By J. N. Hall, M. D., Denver, Colo.............................................543
Scarlet Fever ...............................................113
Smallpox and its Management. By John W. Kenney, M. D., Cooks, New Mexico.............................................550
Some Considerations in the Treatment of the Diseases of Women. By
Some Modern Methods of Local Treatment of the Stomach and the Intestines. By H. Leonards, M. D., New Braunfels, Texas.........604
Some Problems Solved. By R. W. Lowe, M. D., Ridgefield, Conn..... 80
Daniel Parker, M. D ......................................... 30
P. Halsted Fairchild, M. D., New York.........................306
Stab Wound of the Thoracic Duct—Recovery.......................109
Stone in the Urinary Bladder—Report of a Case. By A. N. Denton, M. D., Austin..........................................363
The Dentist's Burden. By F. S. Caspar, D. D. S., Austin, Texas.....693

The Development of Serum Therapy. By Chas. T. M'Clintock, Detroit, Mich ....................................................166
The Differential Diagnosis between Dengue and Yellow Fever, with some Account of the Epidemic of 1897 in Texas. By H. A. West, M. D., Galveston....................................................227
The Doctor as a Peacemaker. By J. O. Scott, M. D., Sherman, Texas. 55
The Importance of Applying a Proper Splint in Fractures of the Patella ....................................................259
The Invisible Enemy and Our Methods of Defense. By M. M. Smith, M. D., Austin, Texas....................................................615
Then and Now: Reminiscent. By D. M. Reagan, M. D., Kyle........360
The Philosophy of Crime and Pauperism, with Suggestions of Possible Methods of Prevention. By F. E. Daniel, M. D.................... 1
The Treatment of Burns. By M. M. Pool, M. D., El Campo..........352
The Treatment of Surgical Shock by Saline Infusions. J. Shelton Horseley, M. D., El Paso, Texas...............................539
The Marriage of the Unfit. Harry Campbell, M. D., B. S. Lond., F. R. C. P. Lond., Physician to the Northwest London Hospital..........172
The Value of Texas Hot Waters. By H. C. Black, M. D., Waco, Texas..623
Tonsilitis—Acute and Chronic....................................558
Treatment of Typhoid Fever. By T. M. Wilson, M. D., Thornton, Texas.302
Two Interesting Cases Reported at the Annual Meeting of the West Texas Medical Association, held in San Antonio, Texas, October 27, 1898. By Alfred C. McDaniel, M. D., San Antonio, Texas.........255

Abstracts and Selections..........................375, 420, 192-212

Abstracts and Selections—

Advances in Our Knowledge of Typhoid Fever.....................701
A New Operation for Hernia. By Emory Lanphear, M. D., Ph. D., St. Louis, Mo. ....................................................697
"Christian Science" and the Practice of Medicine...................326
Death in Crape....................................................330
Let Him Get Well..................................................702
Manufacture of Anti-Diphtheritic Serum.........................328
Memorial to O'Dwyer..............................................698
Observations Upon the Treatment of Some Cases of Neurasthenia. By Jerome K. Bauduy, M. D.......................................... 86
Proceedings of the St. Louis Medical Society...................... 95
Result of the Serum Treatment of Diphtheria in Russia.............329
The Illinois Bill to Regulate the Practice........................315
The Libel Suit of William Smith, Osteopathist.....................327
The Paris Exposition ..............................................699
The Renal Form of Enteric Fever. By J. C. Wilson, M. D., Philadelphia ....................................................320
The Treatment of Carbuncles. By Milton P. Creel, M. D., Central City, Kentucky ....................................................694
When a Feller is Off'n His Feed....................................700

Society Notes—
American Medical Association...................................... 79
American Medical Association......................................561
Annual Meeting of the Western Texas Medical Association...........273
Austin District Medical Society...................................508
Brazos Valley Medical Association.................................271
Communication from Committee on Medical Legislation, Texas State Medical Association..............................................119
Dr. R. M. Swearingen—Resolutions of Respect.—Lamar County Medical Society ....................................................184
His Fiftieth Anniversary.......................................... 76
Meeting of the Western Texas Medical Association..................559
Mississippi Valley Medical Association—Announcement............. 80
Mississippi Valley Medical Association........................184-190

New York Academy of Medicine..................................63
New York Academy of Medicine..................................261
New York Academy of Medicine..................................369
New York Academy of Medicine..................................495
New York Academy of Medicine..................................564
Postponement of the Third American Medical Congress..............312
Requirements for the Degree in Medicine as Adopted by the American Medical Association..............................................190
Richmond Academy of Medicine and Science........................75
Richmond Academy of Medicine and Surgery........................492
South Texas Medical Association..................................635
The Plague in Vienna..............................................274
The Brazos Valley Medical Association............................632
The Southern Surgical and Gynecological Association..............268
The Western Ophthalmologic and Oto-Laryngologic Association......505
Texas Association of Railway Surgeons—Announcement............62
Texas State Medical Association............................506, 419
Texas State Medical Association—Thirty-First Annual Meeting......627
To Members of the Texas State Medical Association................635
Tri-State Medical Society.....................................191-192
United Confederate Veteran's Association.........................633
Western Ophthalmological and Otorlaryngologic Association.........367
Wyoming State Medical Society.....................................311

MEDICAL NEWS AND MISCELLANY......40-44, 100, 153, 155, 588, 658, 215, 216, 252, 532, 341, 403, 461, 715, 718.

BOOKS AND MAGAZINES......44-48, 155, 159, 592, 659, 101, 215, 216, 284, 343, 407, 460, 718-722.

CORRESPONDENCE—

The Anti-Vivisection Bill in Congress............................311

EDITORIAL—

Absolute Quarantine...............................................399
A Fable (After Aesop)..............................................458
A High Handed Outrage.............................................531
An Epileptic Colony for Texas.....................................712
A New Luminary in the East—Pointers for Osler..................703
An Offensive Clause...............................................711
A Subscriber......................................................214
A. U. S. Patent on Antitoxin......................................148
Behring in the Baby Act...........................................280
Canned Meat and Typhoid Fever....................................458
Conference of the Southern Boards of Health......................279
Death of Dr. Hamilton.............................................391
Death of Dr. Renfro...............................................38
Death of Dr. Rutherford...........................................587
Death of Dr. Swearingen...........................................144
Death of Mr. Parke................................................588
Dr. A. B. Gardner, President Texas State Medical Association.......655
Dr. Daniel Du Pre.................................................341
Dr. R. T. Knox....................................................275
Dr. Tebault's Paper...............................................657
Dr. Wilson's Paper................................................339
During October....................................................215
Error.............................................................215
In Darkest England................................................333
Lambert and Listerine.............................................588
Meningitis........................................................530

Morphine Poisoning .....214
National Sanitary Legislation.....331
Our Houston Contemporary.....100
Our Saving Solons .....714
Recognition of Homeopaths and the Code of Medical Ethics.....141
Repeal of the Tax on Doctors.....714
Restrictive Medical Legislation..... 35
"Salus Populi" not "Suprema Lex" in Texas.....587
Smallpox .....460
Some Dont's of Our Own..... 39
State Board of Health Bill.....525
State Health Officer of Texas.....152
State Health Officer's Report.....403
State Lunatic Asylum, Austin.....529
Straight Talk .....152
Texas "Sanitation" by Force and Arms.....581
Texas' Sin of Omission—Her Sanitary Needs.....392
The A. M. A. Meeting in Denver..... 98
The Adulterated Food Law in Texas a Dead Letter.....709
The Austin District Medical Society.....401
The Death of Col. Waring.....282
The Eastern Pest—"Bubonic Plague".....281
The Japanese.....532
The Late Epidemics in Louisiana and Mississippi.....276
The New Treatment for Consumption..... 39
The Old Doctor on "Embalmed Beef" and Things: Homeopathy to the Breach—A Story of a Cock and Bull.....455
The Quarantine Act.....399
The Quarantine Appropriations.....713
The San Antonio Meeting Texas State Medical Association.....584
The San Antonio Meeting.....645
The Standpoint of the Other Fellow.....459
The State Medical Association Bill.....531
The Texas State Medical Association.....402
The West Texas Medical Association.....340
The Yellow Fever Situation.....213
That Woodbridge Treatment for Typhoid Fever..... 39
That Woodbridge Treatment for Typhoid Fever..... 97
To Administer Quarantine.....400
We Extend Our Condolence.....714
Yellow Fever Disarmed and Defied.....150

